# Adding age and sex to improve adverse outcome prediction in suspected infection: validation of a modified National Early Warning Score in the emergency department

**DOI:** 10.1097/MEJ.0000000000001363

**Published:** 2026-08-24

**Authors:** Oren Turgman, Catalien M. Boelens, Michiel Schinkel, Willem Joost Wiersinga, Tom van der Poll

**Affiliations:** a Center for Infection and Molecular Medicine, Amsterdam Institute for Infection and Immunity; b Division of Infectious Diseases, Department of Medicine, Amsterdam UMC, University of Amsterdam, Amsterdam, The Netherlands

Sepsis is a life-threatening organ dysfunction caused by a dysregulated host response to infection [1,2]. Early recognition and treatment are essential but remain challenging because of heterogeneous clinical presentations and underlying conditions [3,4]. Several clinical scores have been developed to identify patients at risk of deterioration; however, they show limited sensitivity or specificity for predicting adverse outcomes in emergency department (ED) patients with infection [5]. Age and sex impact the incidence and mortality of sepsis [6,7].

To evaluate whether adding age and sex to the National Early Warning Score (NEWS) [8] improves prediction of 30-day mortality or ICU admission among adults with suspected infection in the ED, we conducted a retrospective multicenter cohort study including adults (≥18 years) attending the Amsterdam University Medical Center (UMC) EDs with a suspected or confirmed infection between January 2016 and 2021. Suspected infection was defined as the initiation of antibiotic therapy or the collection of a blood culture during the ED stay.

We compared the modified NEWS (mNEWS), which combines age, sex, and original NEWS parameters (respiratory rate, oxygen saturation, heart rate, systolic blood pressure, temperature, Alert, Verbal, Pain, Unresponsive score, and supplementary oxygen; Supplementary Table S1, Supplemental digital content 1, https://links.lww.com/EJEM/A563) with NEWS, the modified Early Warning Score (MEWS) [9], systemic inflammatory response syndrome (SIRS) criteria [10], and quick Sequential Organ Failure Assessment (qSOFA) score [1]. Missing data were addressed by multiple imputation using chained equations using variables listed in Supplementary Table S2, Supplemental digital content 1, https://links.lww.com/EJEM/A563. A sensitivity analysis was performed with a subgroup of patients with less than 3 missing mNEWS components. A second sensitivity analysis included only cases with no missing mNEWS components. Erroneous values were removed using plausible ranges of clinical variables (Supplementary Table S3, Supplemental digital content 1, https://links.lww.com/EJEM/A563). Validation was performed using the MIMIC-IV-ED database (Beth Israel Deaconess Medical Center, Boston, USA; 2011–2019) using the same inclusion criteria. MEWS and SIRS were unavailable in this validation dataset. Only complete cases (no missingness for scoring variables) were used for the validation analysis. The primary composite outcome was 30-day mortality or ICU admission. Receiver operating characteristic (ROC) curves and area under the curve (AUC) values were generated with 2000 bootstrap replicates. Optimal cut-off values were determined using the Youden index. AUCs were compared using DeLong’s test. Two-sided *P* < 0.05 was considered statistically significant. Formal review was waived by the Amsterdam UMC Ethics Committee (IRB00002991; case 2020.486).

A total of 8576 unique ED visits were identified in the Amsterdam UMC cohort (Supplementary Figure S1, Supplemental digital content 1, https://links.lww.com/EJEM/A563) and 93 077 in the MIMIC-IV-ED cohort. Patients in the latter cohort were younger (median 53 vs. 64 years) with lower disease severity and 30-day mortality (1.9 vs. 7.7%) (Supplementary Table S4, Supplemental digital content 1, https://links.lww.com/EJEM/A563). For 5319 Amsterdam UMC ED visits, fewer than three mNEWS components were missing (Supplementary Table S5, Supplemental digital content 1, https://links.lww.com/EJEM/A563). These patients were older (median 66 vs. 60 years), admitted to the hospital and ICU more, and had higher 30-day mortality (9.1 vs. 5.3%) (Supplementary Table S6, Supplemental digital content 1, https://links.lww.com/EJEM/A563). For the whole Amsterdam UMC cohort, the risk of adverse outcome increased with increasing mNEWS (Supplementary Figure S2, Supplemental digital content 1, https://links.lww.com/EJEM/A563). An optimal cut-off of eight resulted in a sensitivity of 0.67 (95% confidence interval: 0.64–0.69) and specificity of 0.65 (0.64–0.66) (Youden index 0.32; Supplementary Table S7, Supplemental digital content 1, https://links.lww.com/EJEM/A563).

The AUC for mNEWS for predicting adverse outcome in the Amsterdam UMC cohort was 0.71 (0.70–0.73); in the MIMIC-IV-ED cohort, it was 0.75 (0.74–0.75) (Fig. 1; Supplementary Table S8, Supplemental digital content 1, https://links.lww.com/EJEM/A563). Within the Amsterdam UMC, mNEWS outperformed NEWS [0.69 (0.67–0.71)], MEWS [0.66 (0.64–0.68)], qSOFA [0.61 (0.60–0.63)], and SIRS [0.56 (0.54–0.57)] (Fig. 1; Supplementary Table S8, Supplemental digital content 1, https://links.lww.com/EJEM/A563). ROC curves are shown in Supplementary Figure S3, Supplemental digital content 1, https://links.lww.com/EJEM/A563. The improved discriminative performance of mNEWS compared with NEWS, the next best-performing score across cohorts, remained consistent in sensitivity analyses restricted to the Amsterdam UMC cohort with less than 3 missing mNEWS components (*n* = 5319; *P* = 0.019) and in the complete-case analysis (*n* = 2232; *P* < 0.001) (Supplementary Tables S8 and S9, Supplemental digital content 1, https://links.lww.com/EJEM/A563). Likewise, in MIMIC-IV-ED, mNEWS [AUC: 0.75 (0.74–0.75)] showed higher discriminative performance than NEWS [0.68 (0.68–0.69)] and qSOFA [0.61 (0.61–0.62)] (*P* < 0.001) (Supplementary Table S9, Supplemental digital content 1, https://links.lww.com/EJEM/A563; Fig. 1). In both cohorts, mNEWS also best predicted 30-day mortality, and in MIMIC-IV-ED, ICU admission (Supplementary Table S8, Supplemental digital content 1, https://links.lww.com/EJEM/A563). Across cohorts, all scores demonstrated higher AUCs in MIMIC-IV-ED, likely reflecting differences in case-mix and outcome distribution (Fig. 1, Supplementary Table S8, Supplemental digital content 1, https://links.lww.com/EJEM/A563).

**Fig. 1:**
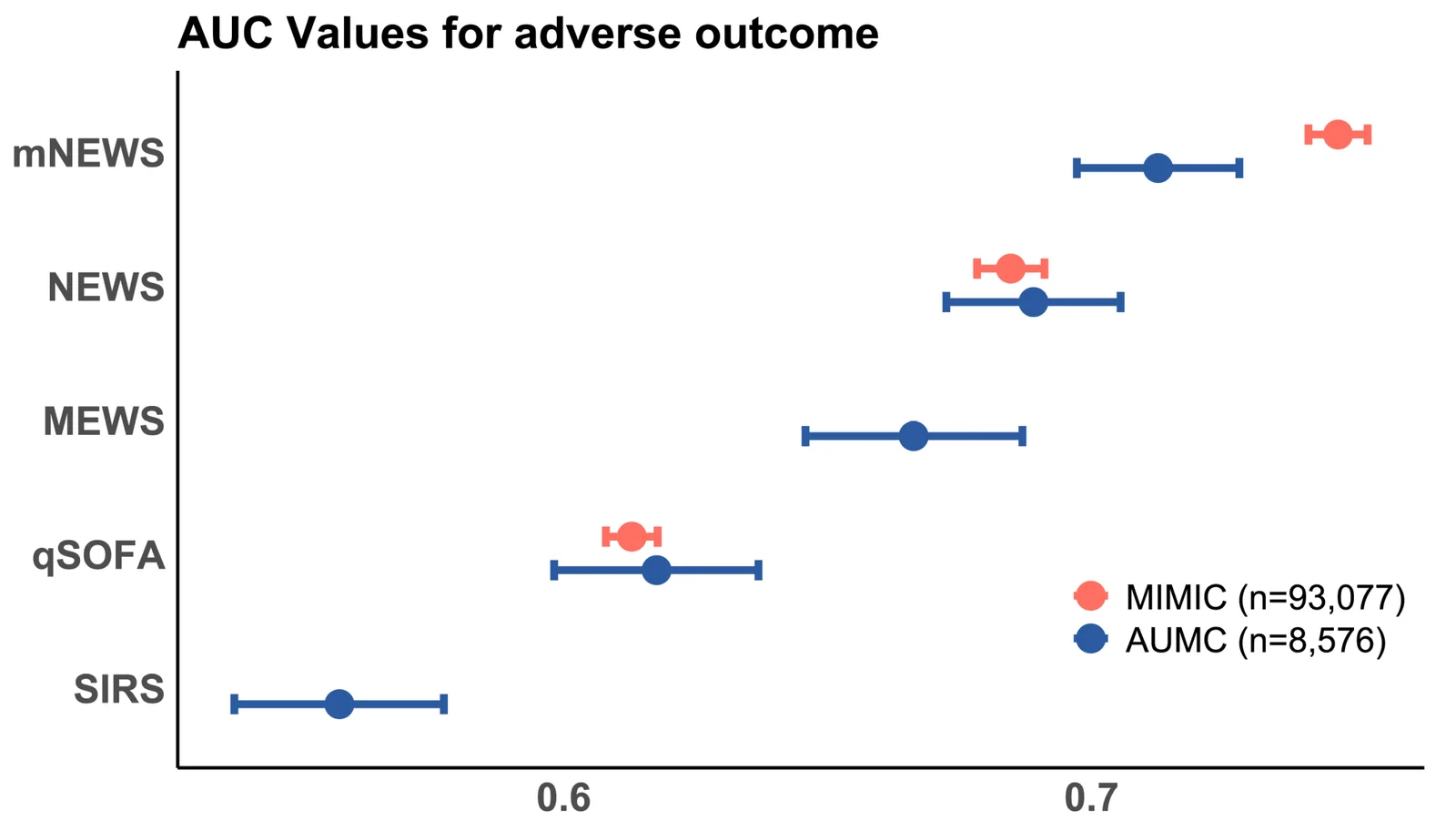
Comparison of AUC values for the mNEWS and other scores in the Amsterdam UMC and MIMIC-IV-ED cohorts. Error bars indicate 95% confidence intervals. In both datasets, AUC values for adverse outcome, defined as mortality within 30 days or ICU admission during hospital stay, are displayed. The mNEWS showed improved discrimination compared with other scores measured (*P* values in Supplementary Table S9, Supplemental digital content 1, https://links.lww.com/EJEM/A563). AUC, area under the curve; MEWS, modified Early Warning Score; mNEWS, modified National Early Warning Score; qSOFA, quick Sequential Organ Failure Assessment; SIRS, systemic inflammatory response syndrome.

This study is the first to assess the value of adding age and sex to an existing clinical score (NEWS) for predicting adverse outcomes in ED patients with suspected infection. The mNEWS demonstrated a modest but statistically significant improvement in prognostic accuracy when compared with not only NEWS but also MEWS, qSOFA, and SIRS. This investigation also demonstrates that missing values in vital parameters, although common in the ED, did not materially affect the improved performance of the mNEWS. The improved performance of the mNEWS across two large but distinct (in terms of disease severity) cohorts adds to the robustness of these results.

Thirty-day mortality was higher in the Amsterdam UMC cohort, whereas ICU admission rates differed only marginally compared with MIMIC-IV-ED. Possible explanations include differences in case-mix and healthcare system practices, such as lower ICU admission thresholds in the MIMIC-IV-ED cohort, differences in escalation practices, and end-of-life decision-making. These findings highlight that ICU admission is a practice-sensitive outcome and may not be directly comparable across healthcare systems. For this reason, we also reported mortality separately in addition to the composite outcome.

Our study has limitations. Measurement bias may exist if vital signs were recorded selectively in sicker patients. Our analyses did not include data from nonacademic hospitals. Since the inclusion period of the Amsterdam UMC cohort partly encompassed the coronavirus disease 2019 (COVID-19) pandemic, a small part of the included patients in that cohort likely had COVID-19. This total proportion of COVID-19 patients over the 5 inclusion years might even be representative of future ED cohorts. In the MIMIC-IV-ED cohort, no COVID-19 patients were included since inclusion ended in 2019.

In this study, the mNEWS outperformed all available scores in two large cohorts enrolled in the Netherlands and the USA. Including age and sex in NEWS offers a simple, implementable improvement for risk stratification of infected ED patients.

## Acknowledgements

O.T. was supported by a grant from the Dutch Ministry of Economic Affairs & Health Holland (DETECT-SEPSIS).

Study design, analysis, and interpretation of results and manuscript preparation: O.T. Study design, analysis, and interpretation of results, manuscript preparation: C.M.B. Data collection, interpretation of results: M.S. W.J.W., and T.v.d.P. Principal investigators, study design, interpretation of results and manuscript preparation: W.J.W. and T.v.d.P. All authors reviewed the results and approved the final version of the manuscript.

### Conflicts of interest

There are no conflicts of interest.

## Supplementary Material

## References

[1] Singer M Deutschman CS Seymour CW Shankar-Hari M Annane D Bauer M. The third international consensus definitions for sepsis and septic shock (Sepsis-3). JAMA 2016; 315:801–810. 26903338 10.1001/jama.2016.0287 PMC4968574

[2] Rudd KE Johnson SC Agesa KM Shackelford KA Tsoi D Kievlan DR. Global, regional, and national sepsis incidence and mortality, 1990-2017: analysis for the Global Burden of Disease Study. Lancet 2020; 395:200–211. 31954465 10.1016/S0140-6736(19)32989-7 PMC6970225

[3] Evans L Rhodes A Alhazzani W Antonelli M Coopersmith CM French C. Surviving sepsis campaign: international guidelines for management of sepsis and septic shock 2021. Intensive Care Med 2021; 47:1181–1247. 34599691 10.1007/s00134-021-06506-y PMC8486643

[4] Wiersinga WJ van der Poll T. Biological drivers of the host response in sepsis. Thorax 2026; 81:396–403. 40603006 10.1136/thorax-2024-222012

[5] Sabir L Ramlakhan S Goodacre S. Comparison of qSOFA and Hospital Early Warning Scores for prognosis in suspected sepsis in emergency department patients: a systematic review. Emerg Med J 2022; 39:284–294. 34404680 10.1136/emermed-2020-210416

[6] Martin GS Mannino DM Moss M. The effect of age on the development and outcome of adult sepsis. Crit Care Med 2006; 34:15–21. 16374151 10.1097/01.ccm.0000194535.82812.ba

[7] Xu J Tong L Yao J Guo Z Lui KY Hu X. Association of sex with clinical outcome in critically ill sepsis patients: a retrospective analysis of the large clinical database MIMIC-III. Shock 2019; 52:146–151. 30138298 10.1097/SHK.0000000000001253 PMC6687414

[8] Royal College of Physicians. National Early Warning Score (NEWS): standardising the assessment of acute-illness severity in the NHS. Report of a working party. RCP; 2012.

[9] Subbe CP Kruger M Rutherford P Gemmel L. Validation of a modified Early Warning Score in medical admissions. QJM 2001; 94:521–526. 11588210 10.1093/qjmed/94.10.521

[10] Bone RC Balk RA Cerra FB Dellinger RP Fein AM Knaus WA. Definitions for sepsis and organ failure and guidelines for the use of innovative therapies in sepsis. The ACCP/SCCM Consensus Conference Committee. American College of Chest Physicians/Society of Critical Care Medicine. Chest 1992; 101:1644–1655. 1303622 10.1378/chest.101.6.1644

