# Supplementary material for: Adding age and sex to improve adverse outcome prediction in suspected infection: validation of a modified National Early Warning Score in the emergency department
Source: Eur J Emerg Med. 2026 Aug 24;33(5):362–4. doi: 10.1097/MEJ.0000000000001363 (PMC13506199; doi:10.1097/MEJ.0000000000001363)
Supplement: Supplementary file 1 [file ejem-33-362-s001.docx]

Supplementary Digital Content

Adding age and sex to improve adverse outcome prediction in suspected infection: validation of a modified National Early Warning Score in the emergency department

# Table of Contents

Supplementary figure 1. Flow diagram of patient inclusion in the Amsterdam UMC cohort	2

Supplementary figure 2. Probability of adverse outcome by mNEWS in the Amsterdam UMC cohort	3

Supplementary figure 3. ROC curves of early warning scores for adverse outcome prediction across cohorts	4

Supplementary table 1. Components and scoring of the mNEWS	5

Supplementary table 2. Variables included in the Multiple Imputation by Chained Equations (MICE) model	6

Supplementary table 3. Plausible physiological and laboratory value ranges used for data cleaning	7

Supplementary table 4. Baseline characteristics of the Amsterdam UMC and MIMIC-IV-ED cohorts	8

Supplementary table 5. Distribution of missing mNEWS components in the Amsterdam UMC cohort	9

Supplementary table 6. Clinical cohort characteristics stratified by missingness of mNEWS components	10

Supplementary table 7. Performance of mNEWS thresholds in the Amsterdam UMC cohort	11

Supplementary table 8: Discriminative performance of early warning scores across outcomes and cohorts	12

Supplementary table 9. Pairwise comparisons of AUCs using DeLong’s test across cohorts	13

References	13

### **Supplementary Figure 1.** Flow diagram of patient inclusion in the Amsterdam UMC cohort

### **Supplementary Figure 2.** Probability of adverse outcome by modified National Early Warning Score (mNEWS) in the Amsterdam UMC cohort

Probability of the composite adverse outcome (30-day mortality or ICU admission during hospitalization) across modified NEWS (mNEWS) score categories in the Amsterdam UMC cohort (n = 8,576). Bars represent the number of patients within each mNEWS score category. The line indicates the proportion of patients experiencing the adverse outcome, demonstrating an increasing risk with higher mNEWS scores.

### **Supplementary Figure 3.** Receiver operating characteristic (ROC) curves of early warning scores for prediction of adverse outcomes across cohorts

Adverse outcome defined as 30-day mortality or ICU admission during hospitalization. Area under the curve (AUC) values and 95% confidence intervals are reported in Supplementary Table 8.

| **Supplementary Table 1**: Components and scoring of the mNEWS | | | | | | | | | |
| --- | --- | --- | --- | --- | --- | --- | --- | --- | --- |
| Score | 3 | 2 | 1 | 0 | 1 | 2 | 3 | 4 |  |
| Respiratory rate (breaths/minute) | <9 |  | 9-11 | 12-20 |  | 21-24 | >24 |  |  |
| Oxygen saturation (%) | <92 | 92-93 | 94-95 | >95 |  |  |  |  |  |
| Heart rate (beats/minute) | <41 |  | 41-50 | 51-90 | 91-110 | 111-130 | >130 |  |  |
| Systolic blood pressure (mmHg) | <91 | 91-100 | 101-110 | 111-219 |  |  | >219 |  |  |
| Temperature (°C) | <35.1 |  | 35.1-36.0 | 36.1-38.0 | 38.1-39.0 | >39.0 |  |  |  |
| AVPU |  |  |  | Alert |  |  | Confused or not alert |  |  |
| Supplementary oxygen |  |  |  | Air |  | Supplementary oxygen |  |  |  |
| Sex |  |  |  | Female | Male |  |  |  |  |
| Age (years) |  |  |  | 16-49 |  | 50-65 | 66-80 | >80 |  |

*Scoring system for the modified National Early Warning Score (mNEWS). Each physiological variable is assigned a score based on predefined thresholds. The total score is calculated as the sum of all component scores.*

### **Supplementary Table 2**: Variables included in the Multiple Imputation by Chained Equations (MICE) model

| **Predictor variables** | **Output variables** |  |
| --- | --- | --- |
| Age | Heart rate |  |
| Sex | Systolic blood pressure |  |
| C-reactive protein | Respiratory rate |  |
| Thrombocytes | Temperature |  |
| Bilirubin | Saturation |  |
| Lymphocytes | Leukocytes |  |
| Neutrophils | Consciousness |  |
| Monocytes | Supplementary oxygen |  |
| Eosinophils |  |  |
| Basophils |  |  |
| Lactate |  |  |
| Creatinine |  |  |
| Hemoglobin |  |  |
| ALAT |  |  |
| Diastolic blood pressure |  |  |
| Norepinephrine administration |  |  |
| Length of ED stay |  |  |
| Length of hospital stay |  |  |
| Length of ICU stay |  |  |
| Hospital admission |  |  |
| ICU admission |  |  |
| 30-day mortality |  |  |
| In-hospital mortality |  |  |

*Predictor variables were selected based on their anticipated association with the output variables. Output variables were chosen based on the extent of missingness among participants. For imputation 50 datasets and 20 iterations were used. Abbreviations: ED, emergency department; ICU, intensive care unit; ALAT, alanine aminotransferase.*

### **Supplementary Table 3**: Plausible physiological and laboratory value ranges used for data cleaning

|  | Minimum | Maximum |  |
| --- | --- | --- | --- |
| Heart rate (beats/minute) | 1 | 300 |  |
| Systolic blood pressure (mmHg) | 40 | 250 |  |
| Diastolic blood pressure (mmHg) | 40 | 250 |  |
| Temperature (°C) | 28 | 45 |  |
| Respiratory rate (breaths/minute) | 1 | 80 |  |
| Oxygen saturation (%) | 15 | 100 |  |
| C-reactive protein, mg/L | 0.01 | 700 |  |
| Leukocytes, 10⁹/L | 0.001 | 60 |  |
| Thrombocytes, 10⁹/L | 0.1 | 2,400 |  |
| Bilirubin, µmol/L | 2 | 1,000 |  |
| Creatinine, µmol/L | 10 | 2,500 |  |
| Hemoglobin, mmol/L | 1 | 17 |  |
| Alanine aminotransferase, U/L | 4 | 15,000 |  |

*Clinical variables were extracted from electronic health records. To reduce the influence of implausible values due to data entry or measurement errors, clinically plausible ranges were predefined based on prior literature (1, 2). Values outside these ranges were considered erroneous and excluded from analysis.*

**Supplementary Table 4:** Baseline characteristics of the Amsterdam UMC and MIMIC-IV-ED cohorts

|  | Amsterdam UMC cohort (n=8,576) | MIMIC-IV-ED cohort (n=93,077) | SMD | p | Missingness in Amsterdam UMC cohort (%) |
| --- | --- | --- | --- | --- | --- |
| **Demographics** |  |  |  |  |  |
| Age, years (median [IQR]) | 64 [49-74] | 53 [33-69] | 0.44 | <0.001 | 0 |
| Female, n (%) | 3,742 (44) |  |  |  | 0 |
| **Vital parameters** |  |  |  |  |  |
| Heart rate, beats per minute (median [IQR]) | 95 [82-109] | 84 [73-97] | 0.526 | <0.001 | 14.1 |
| Systolic blood pressure, mmHg (median [IQR]) | 129 [113-145] | 133 [119-148] | 0.09 | <0.001 | 15.2 |
| Diastolic blood pressure, mmHg (median [IQR]) | 76 [67-87] | 77 [68-86] | 0.013 | 0.478 | 15.6 |
| Respiratory rate, breaths per minute (median [IQR]) | 22 [18-28] | 18 [16-18] | 0.965 | <0.001 | 44.2 |
| Temperature, degrees Celsius (median [IQR]) | 37.7 [36.8-38.6] | 36.7 [36.4-37.1] | 0.713 | <0.001 | 20.2 |
| Saturation, percentage (median [IQR]) | 95 [93-97] | 99 [97-100] | 0.426 | <0.001 | 44.2 |
| Supplementary oxygen, n (%) | 1,218 (14.2) | 2,233 (2.4) | 0.438 | <0.001 | 0 |
| Impaired consciousness, n (%) | 208 (2.4) | 1,348 (1.4) | 0.246 | <0.001 | 60.2 |
| **Laboratory results (Amsterdam UMC only)** |  |  |  |  |  |
| C-reactive protein, mg/L (median [IQR]) | 61.3 [19.1-140.0] |  |  |  | 9.9 |
| Hemoglobin, mmol/L (median [IQR]) | 7.7 [6.7-8.6] |  |  |  | 9.8 |
| Thrombocytes, 10E9/L (median [IQR]) | 231 [174-308] |  |  |  | 14.3 |
| Leukocytes, 10E9/L (median [IQR]) | 10.3 [7.0-14.6] |  |  |  | 9.2 |
| Creatinine, µmol/L (median [IQR]) | 85 [67-117] |  |  |  | 10.4 |
| Bilirubin, µmol/L (median [IQR]) | 9.3 [6.0-16.0] |  |  |  | 33.7 |
| ALAT, U/L (median [IQR]) | 25 [17-42] |  |  |  | 24.8 |
| **Outcomes** |  |  |  |  |  |
| Hospital admission, n (%) | 5,733 (66.8) | 42,641 (45.8) | 0.434 | <0.001 | 0 |
| Hospital length of stay, days (median [IQR]) | 4 [2-9] | 2 [1-4] | 0.445 | <0.001 | 33.2 |
| ICU admission, n (%) | 773 (9.0) | 7963 (8.6) | 0.016 | 0.153 | 0 |
| 30-day mortality, n (%) | 657 (7.7) | 1768 (1.9) | 0.273 | <0.001 | 0 |

*Data are presented as median [interquartile range] or n (%), as appropriate. ED = emergency department; ICU = intensive care unit; IQR = interquartile range; ALAT = alanine aminotransferase.*

### **Supplementary Table 5**: Distribution of missing modified NEWS (mNEWS) components in the Amsterdam UMC cohort

| Number of missing values | Number of patients |
| --- | --- |
| 0 | 2,232 |
| 1 | 2,229 |
| 2 | 858 |
| 3 | 1,823 |
| 4 | 281 |
| 5 | 470 |
| 6 | 683 |

*Number of patients stratified by the count of missing modified NEWS (mNEWS) variables in the Amsterdam UMC cohort.*

### **Supplementary Table 6**: Clinical cohort characteristics stratified by missingness of mNEWS components

|  | **Cohort with <3 missing components** (n=5,319) | **Cohort with ≥3 missing components** (n=3,257) | **SMD** | **p** |
| --- | --- | --- | --- | --- |
| **Demographics** |  |  |  |  |
| Age, years (median [IQR]) | 66 [53-76] | 60 [44-71] | 0.321 | <0.001 |
| Female, n (%) | 2,301 (43.3) | 1,441 (44.2) | 0.02 | 0.385 |
| **Vital parameters** |  |  |  |  |
| Heart rate, beats per minute (median [IQR]) | 96 [83-110] | 92 [80-105] | 0.184 | <0.001 |
| Systolic blood pressure, mmHg (median [IQR]) | 129 [112-146] | 128 [114-143] | 0.028 | 0.765 |
| Diastolic blood pressure, mmHg (median [IQR]) | 77 [67-88] | 75 [67-85] | 0.105 | 0.004 |
| Respiratory rate, breaths per minute (median [IQR]) | 22 [18-28] | 22 [18-24] | 0.095 | 0.929 |
| Temperature, degrees Celsius (median [IQR]) | 37.7 [36.8-38.5] | 37.9 [37.0-38.6] | 0.13 | <0.001 |
| Saturation, percentage (median [IQR]) | 95 [93-97] | 96 [94-97] | 0.13 | 0.528 |
| **Laboratory results** |  |  |  |  |
| C-reactive protein, mg/L (median [IQR]) | 61.9 [19.0-143.3] | 60.5 [20.0-135.0] | 0.05 | 0.532 |
| Hemoglobin, mmol/L (median [IQR]) | 7.7 [6.7-8.6] | 7.8 [6.8-8.6] | 0.013 | 0.683 |
| Thrombocytes, 10E9/L (median [IQR]) | 232 [174-307] | 231 [173-309] | 0.008 | 0.581 |
| Leukocytes, 10E9/L (median [IQR]) | 10.5 [7.1-14.8] | 10.0 [6.9-14.1] | 0.08 | 0.001 |
| Creatinine, µmol/L (median [IQR]) | 86.0 [68.0-121.0] | 83.0 [65.6-111.0] | 0.044 | <0.001 |
| Bilirubin, µmol/L (median [IQR]) | 9.4 [6.1-16.0] | 9.1 [6.0-15.2] | 0.004 | 0.201 |
| ALAT, U/L (median [IQR]) | 25.0 [17.0-42.5] | 25.0 [17.3-42.0] | 0.04 | 0.472 |
| **Outcomes** |  |  |  |  |
| ED length of stay, hours (median [IQR]) | 4 [3-6] | 4 [3-5] | 0.313 | <0.001 |
| Hospital admission, n (%) | 3,777 (71.0) | 1,956 (60.1) | 0.232 | <0.001 |
| Hospital length of stay, days (median [IQR]) | 5 [2-9] | 4 [2-8] | 0.062 | <0.001 |
| ICU admission, n (%) | 573 (10.8) | 200 (6.1) | 0.167 | <0.001 |
| ICU length of stay, days (median [IQR]) | 1 [1-3] | 1 [0-3] | 0.121 | 0.34 |
| 30-day mortality, n (%) | 483 (9.1) | 174 (5.3) | 0.145 | <0.001 |
| In-hospital mortality, n (%) | 305 (5.7) | 113 (3.5) | 0.108 | <0.001 |

*Abbreviations: ED = emergency department; ICU = intensive care unit; IQR = interquartile range; n = number; CRP = C-Reactive Protein; ALAT = alanine aminotransferase*

### **Supplementary Table 7**: Diagnostic performance of modified NEWS (mNEWS) thresholds for prediction of adverse outcome in the Amsterdam UMC cohort

| Threshold | Sensitivity (95% CI) | Specificity (95% CI) | Positive predictive value (95% CI) | Negative predictive value (95% CI) | Youden index |
| --- | --- | --- | --- | --- | --- |
| 1 | 1.00 (1.00-1.00) | 0.01 (0.00-0.01) | 0.14 (0.14-0.15) | 0.98 (0.89-1.00) | 0.01 |
| 2 | 0.99 (0.99-1.00) | 0.02 (0.02-0.03) | 0.15 (0.14-0.15) | 0.95 (0.91-0.98) | 0.02 |
| 3 | 0.99 (0.98-0.99) | 0.06 (0.06-0.07) | 0.15 (0.14-0.16) | 0.97 (0.95-0.98) | 0.05 |
| 4 | 0.96 (0.95-0.97) | 0.13 (0.13-0.14) | 0.16 (0.15-0.17) | 0.96 (0.94-0.97) | 0.1 |
| 5 | 0.92 (0.90-0.94) | 0.25 (0.24-0.26) | 0.17 (0.16-0.18) | 0.95 (0.94-0.96) | 0.17 |
| 6 | 0.87 (0.85-0.89) | 0.38 (0.37-0.39) | 0.19 (0.18-0.20) | 0.95 (0.94-0.95) | 0.25 |
| 7 | 0.79 (0.76-0.81) | 0.52 (0.51-0.53) | 0.21 (0.20-0.23) | 0.94 (0.93-0.94) | 0.31 |
| 8 | 0.67 (0.64-0.69) | 0.65 (0.64-0.66) | 0.24 (0.23-0.26) | 0.92 (0.91-0.93) | 0.32 |
| 9 | 0.54 (0.51-0.57) | 0.76 (0.75-0.77) | 0.27 (0.26-0.29) | 0.91 (0.90-0.92) | 0.30 |
| 10 | 0.45 (0.42-0.47) | 0.83 (0.83-0.84) | 0.31 (0.29-0.33) | 0.90 (0.89-0.91) | 0.28 |
| 11 | 0.33 (0.30-0.36) | 0.89 (0.88-0.90) | 0.33 (0.30-0.36) | 0.89 (0.88-0.90) | 0.22 |
| 12 | 0.23 (0.21-0.26) | 0.93 (0.93-0.94) | 0.37 (0.33-0.40) | 0.88 (0.87-0.89) | 0.17 |
| 13 | 0.15 (0.13-0.17) | 0.96 (0.95-0.96) | 0.39 (0.34-0.43) | 0.87 (0.86-0.88) | 0.11 |
| 14 | 0.08 (0.07-0.10) | 0.98 (0.97-0.98) | 0.38 (0.33-0.45) | 0.86 (0.86-0.87) | 0.06 |
| 15 | 0.04 (0.03-0.06) | 0.99 (0.99-0.99) | 0.40 (0.32-0.49) | 0.86 (0.85-0.87) | 0.03 |
| 16 | 0.02 (0.01-0.03) | 1.00 (0.99-1.00) | 0.51 (0.37-0.65) | 0.86 (0.85-0.87) | 0.02 |
| 17 | 0.01 (0.01-0.02) | 1.00 (1.00-1.00) | 0.52 (0.31-0.73) | 0.86 (0.85-0.87) | 0.01 |
| 18 | 0.00 (0.00-0.01) | 1.00 (1.00-1.00) | 0.71 (0.29-0.96) | 0.86 (0.85-0.86) | 0.00 |
| 19 | 0.00 (0.00-0.01) | 1.00 (1.00-1.00) | 0.75 (0.19-0.99) | 0.86 (0.85-0.86) | 0.00 |
| 20 | 0.00 (0.00-0.01) | 1.00 (1.00-1.00) | 1.00 (0.29-1.00) | 0.86 (0.85-0.86) | 0.00 |
| 21 | 0.00 (0.00-0.00) | 1.00 (1.00-1.00) | NA | 0.86 (0.85-0.86) | 0.00 |
| 22 | 0.00 (0.00-0.00) | 1.00 (1.00-1.00) | NA | 0.86 (0.85-0.86) | 0.00 |

Sensitivity-specificity-positive predictive value (PPV)-negative predictive value (NPV)-and Youden index across mNEWS score thresholds for predicting the composite outcome of 30-day mortality or ICU admission during hospitalisation in the Amsterdam UMC cohort. Values are presented with 95% confidence intervals where applicable.

**Supplementary Table 8**: Discriminative performance (AUC) of early warning scores across outcomes and cohorts

| **Cohort** | **Outcome** | **Modified NEWS** | **NEWS** | **MEWS** | **qSOFA** | **SIRS** |
| --- | --- | --- | --- | --- | --- | --- |
| Amsterdam UMC (n=8,576) | Mortality | 0.72 (0.70-0.74) | 0.66 (0.64-0.68) | 0.63 (0.61-0.65) | 0.59 (0.57-0.61) | 0.55 (0.53-0.58) |
|  | ICU admission | 0.69 (0.67-0.71) | 0.69 (0.67-0.71) | 0.67 (0.65-0.69) | 0.62 (0.60-0.64) | 0.56 (0.54-0.58) |
|  | Adverse | 0.71 (0.70–0.73) | 0.69 (0.67-0.71) | 0.66 (0.64-0.68) | 0.61 (0.60-0.63) | 0.56 (0.54-0.57) |
| Amsterdam UMC-<3 missing components (n=5,319) | Mortality | 0.71 (0.69-0.73) | 0.67 (0.65-0.70) | 0.63 (0.61-0.66) | 0.61 (0.59-0.64) | 0.57 (0.55-0.59) |
|  | ICU admission | 0.68 (0.66-0.70) | 0.70 (0.67-0.72) | 0.68 (0.66-0.70) | 0.64 (0.62-0.66) | 0.57 (0.55-0.59) |
|  | Adverse | 0.71 (0.69-0.72) | 0.70 (0.68-0.72) | 0.67 (0.65-0.69) | 0.64 (0.62-0.65) | 0.57 (0.55-0.59) |
| Amsterdam UMC-complete-case cohort* | Mortality | 0.74 (0.71-0.78) | 0.70 (0.67-0.74) | 0.63 (0.59-0.68) | 0.66 (0.62-0.70) | 0.57 (0.54-0.60) |
|  | ICU admission | 0.71 (0.68-0.75) | 0.72 (0.69-0.76) | 0.69 (0.65-0.73) | 0.69 (0.65-0.72) | 0.59 (0.56-0.62) |
|  | Adverse | 0.73 (0.71-0.76) | 0.72 (0.69-0.75) | 0.67 (0.64-0.70) | 0.68 (0.65-0.71) | 0.58 (0.56-0.60) |
| MIMIC-IV-ED-complete-case cohort** | Mortality | 0.82 (0.82-0.83) | 0.71 (0.70-0.73) | n/a | 0.64 (0.63-0.65) | n/a |
|  | ICU admission | 0.74 (0.73-0.74) | 0.69 (0.68-0.69) | n/a | 0.61 (0.61-0.62) | n/a |
|  | Adverse | 0.75 (0.74-0.75) | 0.68 (0.68-0.69) | n/a | 0.61 (0.61-0.62) | n/a |

Area under the receiver operating characteristic curve (AUC) with 95% confidence intervals for modified NEWS, NEWS, MEWS, qSOFA, and SIRS across multiple cohorts and outcomes, including 30-day mortality, ICU admission and a composite adverse outcome (30-day mortality or ICU admission during hospitalisation). *Complete case analyses differed by score due to variable-specific missingness: modified NEWS, NEWS and MEWS (n = 2,232), qSOFA (n = 2,365) and SIRS (n = 4,058); **Complete case analyses differed by score due to variable-specific missingness: modified NEWS and NEWS (n=90,782) and qSOFA (n = 93,077); n/a: score not computed in this cohort

### **Supplementary Table 9**: Pairwise comparisons of AUCs using DeLong’s test for the composite adverse outcome across cohorts

|  | Amsterdam UMC cohort | Amsterdam UMC (<3 missing mNEWS components) | Amsterdam UMC (complete cases) | MIMIC-IV-ED cohort |
| --- | --- | --- | --- | --- |
| mNEWS – NEWS | p<0.001 | p=0.019 | p<0.001 | p<0.001 |
| mNEWS – MEWS | p<0.001 | p<0.001 | p<0.001 | NA |
| mNEWS – qSOFA | p<0.001 | p<0.001 | p<0.001 | p<0.001 |
| mNEWS – SIRS | p<0.001 | p<0.001 | p<0.001 | NA |

*P-values from DeLong’s test comparing the area under the receiver operating characteristic curve (AUC) between scoring systems for prediction of the composite adverse outcome (30-day mortality or ICU admission during hospitalization) within each cohort. P-values indicate whether differences in AUC between scores are statistically significant.*

# References

1.	Boerman AW, Schinkel M, Meijerink L, van den Ende ES, Pladet LC, Scholtemeijer MG, et al. Using machine learning to predict blood culture outcomes in the emergency department: a single-centre, retrospective, observational study. BMJ Open. 2022;12(1):e053332.

2.	Schinkel M, Boerman AW, Bennis FC, Minderhoud TC, Lie M, Peters-Sengers H, et al. Diagnostic stewardship for blood cultures in the emergency department: A multicenter validation and prospective evaluation of a machine learning prediction tool. EBioMedicine. 2022;82:104176.
